# Supplementary material for: Activation of Gossypium hirsutum ACS6 Facilitates Fiber Development by Improving Sucrose Metabolism and Transport
Source: Plants (Basel). 2023 Oct 11;12(20):3530. doi: 10.3390/plants12203530 (PMC10610492; doi:10.3390/plants12203530)
Supplement: Supplementary file 1 [file plants-12-03530-s001.zip › plants-2608157-supplementary.pdf]

# Supplementary Table S1: The primer used in this study.

| Primer Name | Sequence (5'-3') |
|---|---|
| *GhACS6.3*-F | CCTGGTTTCAGAGTCGGCAT |
| *GhACS6.3*-R | ATGCTGAGTTTGCGAGGACA |
| *GhACS10.1*-F | AGCCTGCTAGACTTTGCCAG |
| *GhACS10.1*-F | CGATTTCTGCCATGCTCACG |
| *GhMML3*-F | ATCGACCCTGCAACTCACAG |
| *GhMML3*-R | TCGAGGCATTGCGGTTTAGT |
| *GhMML4*-F | TTGAACGATGTGGGAAGAGC |
| *GhMML4*-R | GGAGGTTGAGGGCTTGTGAG |
| *GhMML7*-F | CTGGTTCGTGAGTCCAAGCT |
| *GhMML7*-R | GCCATGCTTTGAGTACGTCG |
| *GhSUT1*-F | TATCAACCCGGCACCAAGTC |
| *GhSUT1*-R | GCCAAATGAAAGCAGCCCAA |
| *GhSUT2*-F | CACCACCACGAAAGCTTGTG |
| *GhSUT2*-R | AGGTTACCGGCATTTCCAGG |
| *GhSWEET2*-F | TCACTGTTCAGTGCGATGCT |
| *GhSWEET2*-R | AGACAAGTCCGAACCCGAAA |
| *GhSWEET5*-F | ATCGGCTCAGCAAGTAGTCG |
| *GhSWEET5*-R | CGGTTCCGTCACCATTGTTG |
| *GhSuSy1*-F | GATTCCACACTTACCGCCCA |
| *GhSuSy1*-R | GATGGCTTCCTGACTGGCTT |
| *GhSuSy3*-F | ATCAGCGGCTGGAAAGAGTC |
| *GhSuSy3*-R | CGTCCTCCGCATAAGTCTCC |
| *GhUBQ7*-F | CGGCAAGCAACTCGAAGATG |
| *GhUBQ7*-R | GACAGCGAGCTTGACCTTCT |

Supplementary Figure S1 Expression profiles of *GhACS*s in leaves, root, stem, torus, stamen, pistil, petal and calycle.

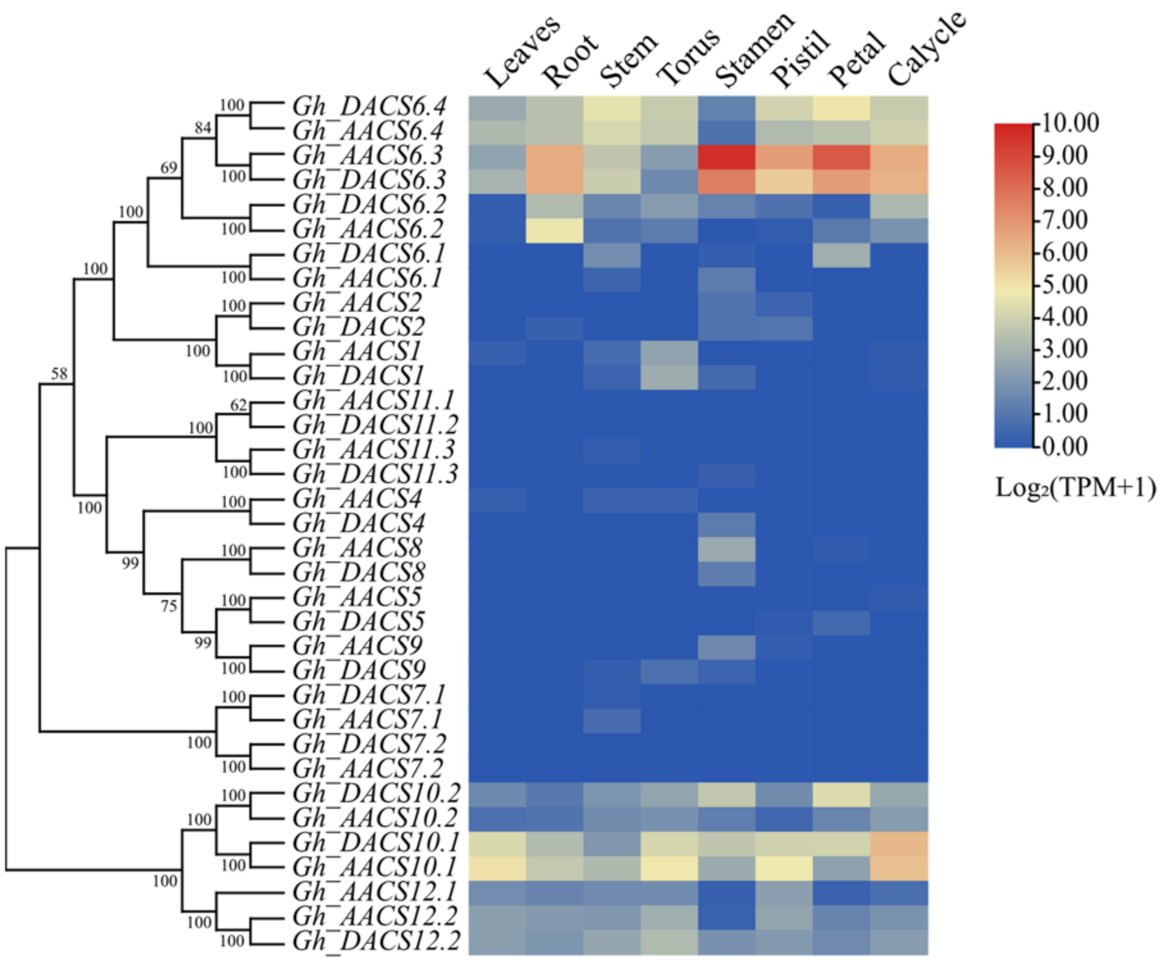

**Supplementary Figure S1:** Expression profiles of *GhACS*s in leaves, root, stem, torus, stamen, pistil, petal and calycle.

Phylogenetic analysis of 35 *GhACSs* in *G. hirsutum* were used to construct the neighbor-joining tree using MEGA 7.0 software. Heatmap generated from RNA-seq data (SRA:PRJNA248163) of *GhACSs* in leaves, root, stem, torus, stamen, pistil, petal and calycle of *G. hirsutum*. Different columns in the figure represent various samples, and rows represent genes.

Supplementary Figure S2: The size of culture ovules with ACC treatment.

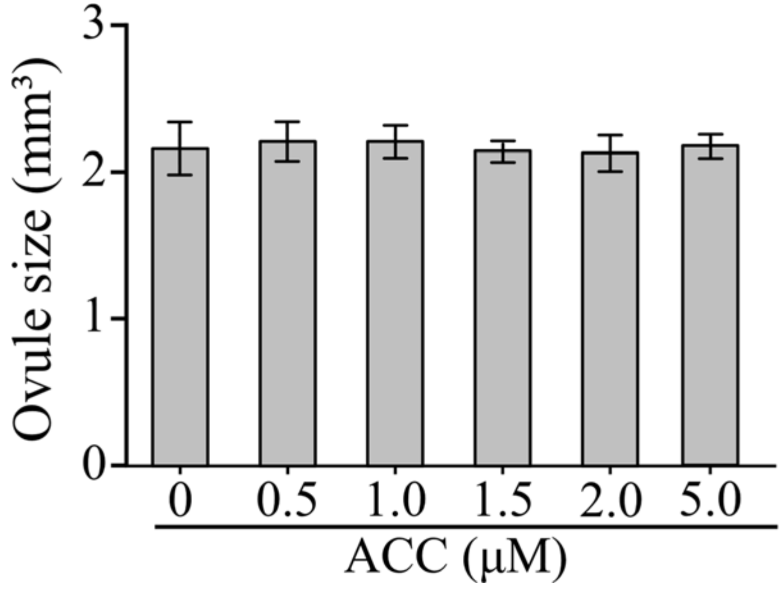


**Supplementary Figure S2:** The size of culture ovules with ACC treatment. Statistics of the size of ovules cultured in vitro for 48 h with 0, 0.5, 1.0, 1.5, 2.0 and 5.0 μM ACC treatment.

Differences between groups were compared using Student's *t-test* ('*', $P < 0.05$, '**', $P < 0.01$). Experiments were repeated five times with similar results, Error bars represent ± SD.

Supplementary Figure S3: Expression profile of *GhSUTs* and *GhSWEETs* in ovules and leaves.

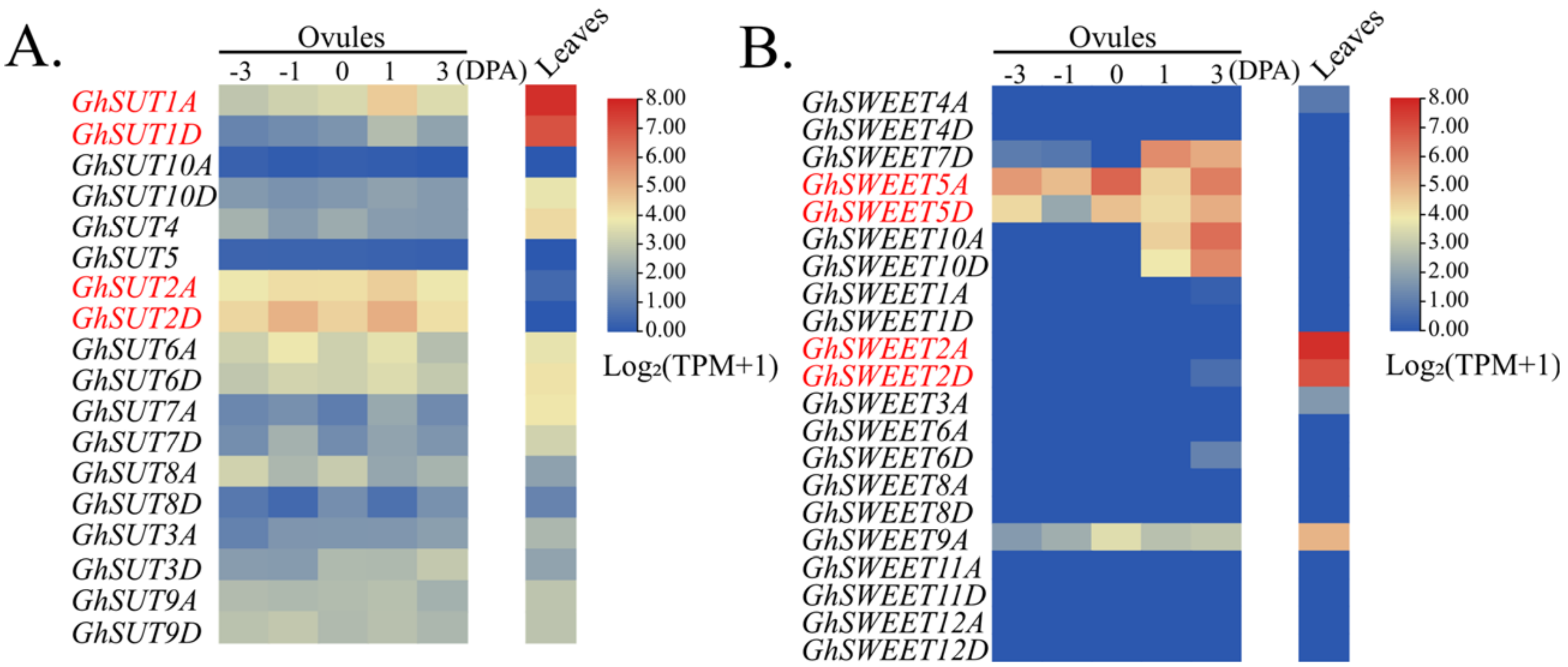


**Supplementary Figure S3** Expression profiles of *GhSUTs* and *GhSWEETs* in ovules during fiber initiation and in leaves. **A and B.** Expression profile of *GhSUTs* and *GhSWEETs* in ovules at -3, -1, 0, 1 and 3 DPA developmental stages and in leaves of *G. hirsutum*. Heatmap generated from RNA-seq data (SRA:PRJNA248163). The color represents the level of expression of the gene ($\log_2$ (TPM+1)) in the sample.

Supplementary Figure S4: Expression profile of *GhSuSys* in ovules and leaves.

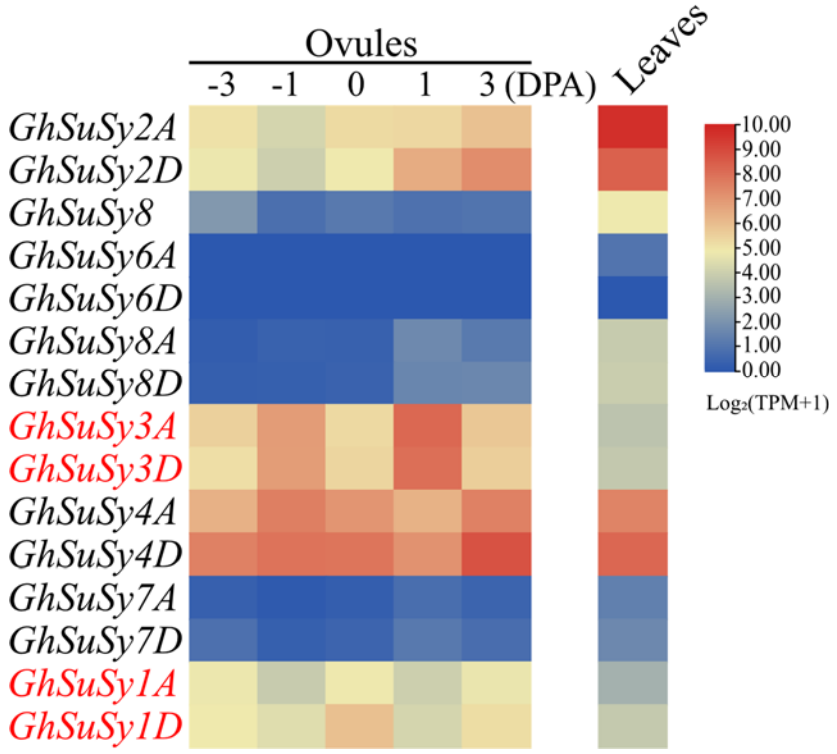


**Supplementary Figure S4:** Expression profile of *GhSuSys* in ovules and leaves during fiber initiation. Heatmap generated from RNA-seq data (SRA:PRJNA248163) of *GhSuSys* in ovules at -3, -1, 0, 1 and 3 DPA developmental stages and in leaves of *G. hirsutum*. Different columns in the figure represent various samples, and rows represent genes. The color represents the level of expression of the gene ($\log^2$ (TPM+1)) in the sample.

Supplementary Figure S5: Fructose content in ovules of the *GhACS6.3*-OE lines.

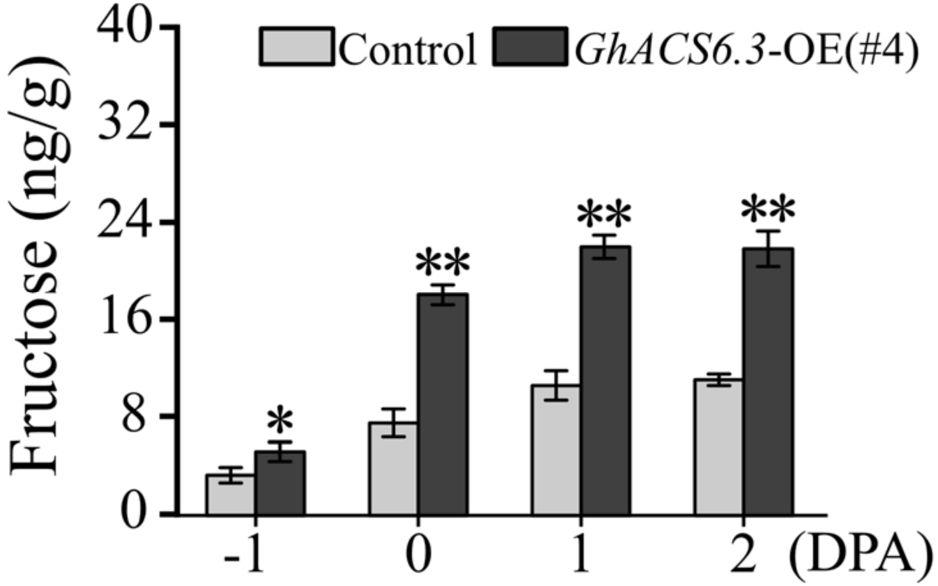


**Supplementary Figure S5:** Fructose content in ovules of the *GhACS6.3*-OE lines.

HPLC analysis fructose in ovules at -1 to 2 DPA development stages of the control and *GhACS6.3*-OE(#4) lines. Differences between groups were compared using Student's *t-test* ('*', $P < 0.05$, '**', $P < 0.01$). Experiments were repeated three times with similar results, Error bars represent ± SD.
